# Difficulties of Diagnosis

**Published:** 1880-11

**Authors:** 


### DIFFICULTIES OF DIAGNOSIS.

The prevalence of a mild grade of fever in the Plaquemine Parish, La., on the Lower Mississippi, during the months of August and September, has furnished another instance of the difficulty of ascertaining reliably the *nature* of certain outbreaks of disease, especially when of limited extent and duration. The fever to which we have alluded in Louisiana, commencing at a time when yellow fever was anxiously watched for, was quickly brought to the notice of the member of the National Board of Health resident in New Orleans. He sent Dr. G. M. Sternberg, of the U. S. A., to investigate the same, and he reported that it was a mild type of genuine yellow fever.

Founded upon this decision, the National Board of Health promptly order.d an appropriation of from $5,000 to $10,000 to be placed at the disposal of the Louisiana State Board of Health, for use in executing necessary measures for preventing the spread of disease. But as the Louisiana State Board regarded the fever in question as one of *malarial* origin, the generous tender of aid from the National Board was declined. At this point the member of the latter board in New Orleans called to his assistance the member of the same board at Memphis, and, after consultation, it was agreed that a committee consisting of Dr. J. Dickson Bruns, of New Orleans, Dr. J. P. Davidson, of the Louisiana State Board of Health, and Surgeon Sternberg, of U. S. A., should visit the infected district and personally make such examination as would enable them to decide more satisfactorily the nature of the prevailing disease.

The results of the investigations of this committee were two reports—one by Surgeon Sternberg, adhering to his previously expressed opinion that the disease was a "mild type of yellow fever;" the other by Drs. Bruns and Davidson, though written by the former, declares as follows: "Of the nature of the fever, without multiplying details, I will simply say that neither in its special features nor in their entirety could I realize a single prominent characteristic of yellow fever." When we remember that all the members of the committee were not only members of the profession in high standing, but thoroughly familiar with yellow fever for years, their arrival at opposite conclusions is somewhat remarkable. It should be stated, however, that, notwithstanding the report of a majority of the committee was to the effect that the disease in Plaquemine Parish and neighboring districts was not yellow fever, the members of the National Board of Health resident in New Orleans and Memphis side with the report of the minority of the committee, and in the *National Board of Health Bulletin* for October 9, declare as follows:

"1. That yellow fever (about one hundred cases) existed between August 1 and September 10 in Plaquemine Parish, La.

"2. That the outbreak had its origin in the immediate vicinity of the Mississippi River Quarantine Station, the first case (August 1, 1880) occurring directly opposite the point where the infected bark 'Excelsior' was detained from July 11 to August 16."

Consequently, all interested in the study of yellow fever, whether sanitarians or practitioners, who look for facts and statistics to the published records of official sanitary organizations, will be likely to take the authority of the National Board as conclusive, and include this fever on the Lower Mississippi in 1880 as an acknowledged prevalence of yellow fever. This affords a fair sample of many of the alleged facts concerning the prevalence of particular diseases to be found in our medical literature, and it gives rise to several important reflections: First, it forces us to suspect that many of the so-called facts concerning the nature, prevalence and prevention of diseases of which we read, rest on imperfectly observed or doubtfully established data. Second, if we assume that Drs. Sternberg, Bemis and Mitchell are correct (and we are not disposed at present to criticise their opinion), we must acknowledge that a prevalence of mild yellow fever has existed over a considerable district of country, beginning in spite of quarantine against importation, and ceasing to spread without internal quarantine or isolation and with but little sanitary interference. But supposing the State Board of Louisiana had accepted the appropriation of the National Board and spent the five or ten thousand dollars in a vigorous isolation and disinfection of every case in the Plaquemine Parish, what would have been the inference? The disease would doubtless have maintained its mild character and disappeared in due time, and would not such a result have been claimed as another triumph of enlightened sanitation over the prevalence of disease? Certainly it would. And yet the claim would have been entirely deceptive or false, as many of those already on record doubtless are. We are not now arguing against the importance of sanitary or preventive medicine, but only trying to realize how easy it is to be deceived, or, more properly, how difficult it is to keep the line of distinction between true *effects* and mere coincidences.

There is, however, another view to be taken of this subject, of no little interest. Those who look over the number of the *Bulletin* to which we have alluded, will see that Drs. Sternberg and Bemis both claim that the disease on the Lower Mississippi, which they call yellow fever, is of a very mild type, causing a very low ratio of mortality. Speaking of his two visits, Dr. Sternberg says: "I have not seen during either visit any case which, *alone*, would enable me to make a positive diagnosis of yellow fever; but from a consideration of all the cases seen by me during my two visits, and of the facts relating to the origin and progress of the epidemic, cannot doubt that this fever is *the mild type* of yellow fever which has been described under various synonyms given in my previous report, and which Blair more properly calls 'yellow fever *simplex*,' to distinguish it from the more malignant type called by him '*gravier*.'" In another place, after alluding to the opinions of Bérenger-Feraud and others, he says: "Such is the resemblance of this form of fever with the first degree of yellow fever that when it is observed sporadically without an epidemic of yellow fever, the doctors of the country say, 'If we were in the time of yellow fever, we would say that it is yellow fever.'" And he adds: "For me, this fever is identical with yellow fever, and only differs in degree from the more severe forms which, because of the fatality which attends them, are known and dreaded by all." If we concede the correctness of Dr. Sternberg's position, that the mild fever of Plaquemine Parish, and the sporadic cases resembling yellow fever that occur in various places within the yellow fever zone almost every year, are really idendical with yellow fever proper, differing only in degree of severity, precisely the same process of reasoning on a parallel series of facts would compel us to regard the ordinary endemic cholera morbus as identical with epidemic cholera; and sporadic erysipelas as differing from the epidemic form only in degree of severity, etc., etc.

But, taking this view, what becomes of the doctrine of the importation of germs, and the spread of these diseases by contagion?

Shall we say that the same identical diseases may be indiginous and non-contagious when in a mild form, and dependent on an imported cause and freely spread by contagion when severe? These are questions which we have long regarded as worthy of the most careful consideration, but which we have not now time to answer.

